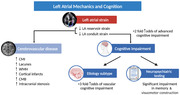# Atrial function: Association with cerebrovascular disease and cognitive dysfunction

**DOI:** 10.1002/alz.088067

**Published:** 2025-01-09

**Authors:** Eugene Tan, Saima Hilal, Siew‐Pang Chan, Ming Ann Sim, Mitchell Kim Peng Lai, Joyce R Chong, Caroline Robert, Hazliza Hazli, Lingli Gong, Josephine Berboso, Narayanaswamy Venketasubramanian, Boon Yeow Tan, Arthur Mark Richards, Christopher Chen, Ling Lieng‐Hsi

**Affiliations:** ^1^ National University Heart Centre Singapore, Singapore, SIngapore Singapore; ^2^ Saw Swee Hock School of Public Health, National University of Singapore and National University Health System, Singapore, Singapore Singapore; ^3^ National University Heart Centre Singapore, Singapore Singapore; ^4^ National University of Singapore, Singapore, singapore Singapore; ^5^ National University of Singapore, Kent Ridge Singapore; ^6^ Yong Loo Lin School of Medicine, National University of Singapore, Singapore Singapore; ^7^ National University of Singapore, Singapore Singapore; ^8^ Raffles Neuroscience Centre, Raffles Hospital, Singapore Singapore; ^9^ St. Luke’s Hospital, Singapore Singapore; ^10^ Christchurch Heart Institute, University of Otago, New Zealand, Christchurch New Zealand; ^11^ Memory, Ageing and Cognition Centre, National University Health System, Singapore Singapore; ^12^ Department of Medicine, Yong Loo Lin School of Medicine, National University of Singapore, Singapore Singapore

## Abstract

**Background:**

The relationships of left atrial (LA) dysfunction with cognition are poorly understood. We investigated the associations of LA function with cognitive impairment, etiologic‐subtype (neurodegenerative vs. vascular[VCI]), neuroimaging markers of cerebrovascular disease (CeVD) and circulating biomarkers, in subjects without AF.

**Method:**

In this prospective cohort study of subjects recruited from memory clinics with brain magnetic resonance imaging (MRI), neuropsychological assessments, circulating biomarker measurements, LA strain (reservoir [LASr], conduit [LAScd], contractile) was determined using 2D speckle‐tracking echocardiography. VCI was defined as vascular dementia or cognitive impairment no dementia(CIND)/Alzheimer’s dementia with significant CeVD, and neurodegenerative defined as CIND/dementia without significant CeVD on neuroimaging.

**Result:**

Among 251 subjects (age 75±8years, 59% female, 178(71%) had cognitive impairment (37% dementia, 14% moderate CIND, 20% mild CIND), of which 58% were VCI and 42% neurodegenerative. LAScd was the only LA strain parameter independently associated with more severe (moderate CIND/dementia vs. mild CIND/no cognitive impairment) grades of cognitive impairment (lowest vs. highest tertile: adjusted odds ratio[AOR] 2.50, p=0.015), and worse MMSE scores, visuomotor construction and memory on neuropsychological testing (p<0.05). LAScd was independently associated with VCI (vs. neurodegeneration) (lowest vs. highest tertile: AOR 3.16, p=0.006), and showed no associations with markers of neurodegeneration, including circulating pTau‐181 and strictly lobar cerebral microbleeds (p>0.05). LAScd and LASr were both associated with increased burden of CeVD including intracranial stenoses, cortical infarcts, lacunes, cerebral microinfarcts, microbleeds and white matter hyperintensities and correlated with cardiac‐specific biomarkers (p<0.05), but LAScd correlated additionally with a broader range of circulating biomarkers reflecting inflammation, neurotrophic processes and neuronal damage (p<0.05).

**Conclusion:**

Reduced LA conduit function was associated with more severe cognitive impairment, primarily due to CeVD. LA strain, particularly LAScd, may be a useful biomarker of cognitive impairment in at‐risk subjects without AF.